# Case report: Scleromyxedema associated with a monoclonal gammapathy: Successful treatment with intravenous immunoglobulins

**DOI:** 10.3389/fimmu.2022.1099918

**Published:** 2023-01-13

**Authors:** Shang-shang Wang, Qin-yi Chen, Lei-hong Xiang

**Affiliations:** Department of Dermatology, Huashan Hospital, Fudan University, Shanghai, China

**Keywords:** sclerodermoid disorders, monoclonal gammapathy, intravenous immunoglobulin therapy, treatment, scleromyxedema

## Abstract

Scleromyxedema is a rare idiopathic fibromucinous disorder characterized by a generalized papular and sclerodermoid cutaneous eruption. Patients often have praraproteinemia and extracutaneous, even lethal, manifestations. Yet the prognostic and therapeutic features of scleromyxedema are poorly documented. High-dose intravenous immunoglobulin (IVIG), used either alone or in conjunction with systemic steroids and/or thalidomide, has been suggested as a first-line treatment. We report the case of a 45-year-old woman diagnosed with scleromyxedema with paraproteinemia that initially did not respond to systemic steroids, retinoids, and thalidomide but greatly improvement in terms of systemic and cutaneous symptoms after treatment with IVIG.

## 1 Introduction

Scleromyxedema is a rare, chronic form of cutaneous mucinosis characterized by numerous, closely spaced waxy papules and diffuse skin induration that usually affects middle-aged adults aged between 30 and 80 years (1). It is almost always accompanied by the presence of a monoclonal gammopathy, typically of the immunoglobulin G (IgG) lambda type (2). Systemic manifestations are common, involving renal, gastrointestinal, pulmonary, cardiovascular, musculoskeletal, or nervous systems and may lead to significant morbidity and mortality (3). Diagnosis is based on (1) clinical findings of a generalized papular and sclerodermoid eruption; (2) a typical histologic triad of mucin deposition, fibrosis, and fibroblast proliferation; (3) a monoclonal gammopathy; and (4) the absence of thyroid disorders (2). There are no standardized treatment guidelines for scleromyxedema as of yet. Therapeutic treatment options include systemic steroids, melphalan, retinoids, cyclophosphamide, high-dose intravenous immunoglobulin (IVIG), thalidomide, and autologous stem cell transplantation (SCT) (4). Here, we reported a case of scleromyxedema in which the patient was initially trialed on steroids, retinoids, and thalidomide without significant improvement, but successfully responded to intravenous immunoglobulin at our clinic.

## 2 Case report

A 45-year-old Chinese female presented with a 2-year history of 2- to 3-mm, generalized, symmetric waxy papules over the face, neck, trunk, and extremities with progressive skin induration. She was previously diagnosed with scleromyxedema at another clinic center based on (1) dermal mucin deposit and fibrosis under microscopy, (2) a monoclonal gammopathy by serum protein electrophoresis, (3) absence of plasma cell of aberrant morphology on bone marrow examination, and (4) an absence of thyroid diseases. She was administered with 30 mg of oral acitretin per day for 4 months without improvement, and then switched to 16 mg of oral methylprednisolone, together with 50 mg of thalidomide, per day for 6 months. However, the patient’s skin swelling and thickening worsened during this 6-month period. She also developed systemic symptoms, such as generalized fatigue, limited joint mobility, dizziness, and dysphagia. She was otherwise (excepting a medical history of cataract and mild anemia) in a state of good health.

**Figure 1:**
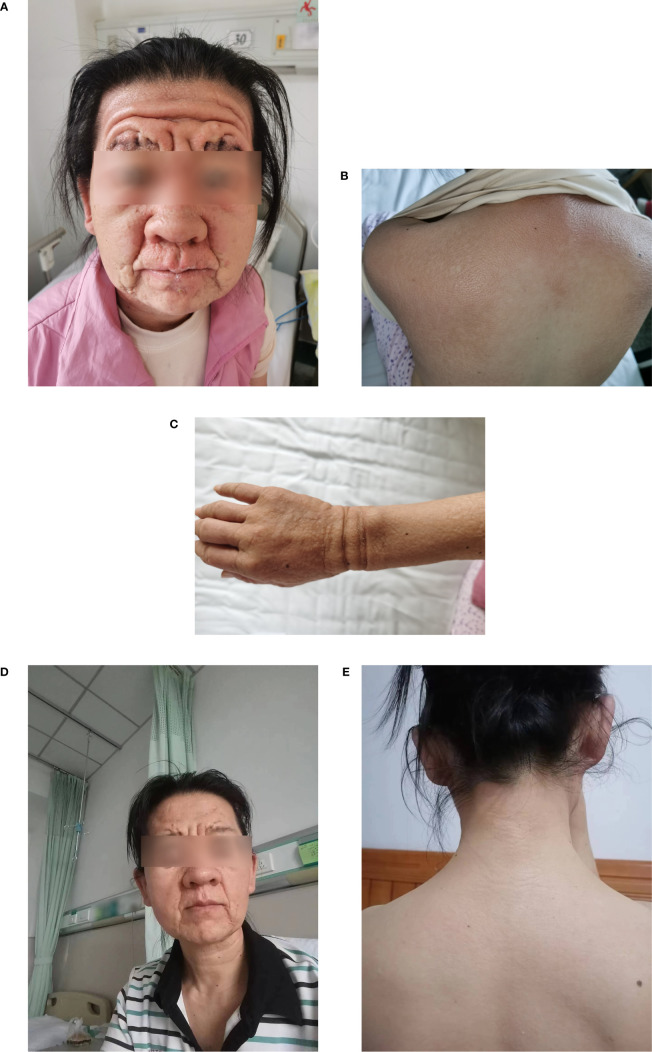
**(A–C)** Cutaneous manifestations before IVIG treatment; **(D, E)** after six courses of IVIG treatment. IVIG, high-dose intravenous immunoglobulin.

**Figure 2:**
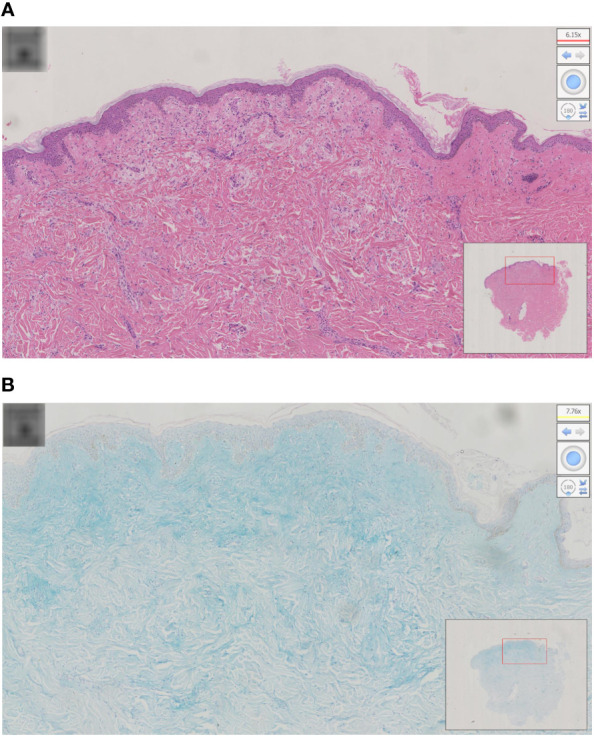
**(A)** Histopathology of scleromyxedema with the typical triad of mucin, fibroblast proliferation, and fibrosis; **(B)** alcian blue staining was positive in the upper and mid dermis.

Physical examination revealed a leonine face due to thickened skin folds and furrows. Diffuse, skin-colored, 2- to 3-mm, firm, and waxy papules in a linear arrangement coalesced into indurated plaques over the trunk and extremities (**Figures 1A–C**). A skin biopsy found evidence of irregular fibrosis and fibroblast proliferation with interstitial mucin deposits (**Figure 2A**). Alcian blue staining was positive in the upper and mid dermis (**Figure 2B**). These histopathologic findings were consistent with the diagnosis of scleromyxedema.

Laboratory results were significant for mild anemia (hemoglobin 106g/L) and showed an increased erythrocyte sedimentation rate of 22 mm/L h (normal 0–15mm/L h). Immunoelectrophoresis and immunofixation showed monoclonal spikes IgG Lambda (λ) (6.26 g/L) in blood samples, but not in urine samples. Free thyroxin, thyroid stimulating hormone levels, serum lipid levels, hepatic and renal function, and glucose levels were within normal limits. Screenings for antinuclear antibodies, human immunodeficiency virus, hepatitis B, hepatitis C, and malignancy were negative. Results were non-significant for the chest radiography, abdominal ultrasound, electrocardiography, echocardiography, electromyography, and bone marrow biopsy examinations. The patient was administered with intravenous immunoglobulin (IVIG) therapy at a dose of 2g/kg/month (over a 5-day treatment cycle) for 6 months. She tolerated this treatment well and her skin symptoms and muscle weakness, dysphagia, and fatigue were significantly improved after six cycles of treatment (**Figures 1D, E**). Serum protein electrophoresis showed a progressive decrease in IgG λ. The patient is still receiving treatment and attending follow-up visits.

## 3 Discussion

Scleromyxedema is a chronic and progressive disease, manifesting physically in the form of waxy papules and diffuse skin induration, usually in association with systemic involvement and paraproteinemia (1). Scleromyxedema usually affects middle-aged adults with no predominance based on race or sex (accurate incidence data are not yet available) (5). Dermal mucin deposits, increased fibroblast proliferation, and marked collagen deposition are the disease’s key histologic features (2). The exact etiology of this disease remains unclear. *In vitro* studies show that serum extracts from patients could stimulate fibroblast proliferation and glycosaminoglycans synthesis, indicating the induction of abnormal fibrosis by circulating cytokines, such as interleukin-1, tumor necrosis factor-α, and transforming growth factor-β (6). The cutaneous manifestations of scleromyxedema are 2- to 3-mm, firm, waxy, slightly reddish or skin-colored, dome-shaped, or flat papules with widespread distribution (1). The most commonly affected areas are the face, neck, distal forearms, and hands, sparing the palms, scalp, and mucous membranes. Leonine faces may be observed in patients with diffuse mucin deposition within the glabella. The thickening of the proximal interphalangeal joints with central depressions, and deep furrows on the back or extremities, are also common physical symptoms of scleromyxedema, and are known as doughnut signs and Shar-Pei signs, respectively (6). The systemic complications caused by scleromyxedema can be lethal in some cases. The most common extracutaneous manifestation is plasma cell dyscrasia with monoclonal gammopathy of undetermined significance, as shown in our case (5). Other complications include involvement of the neurologic, rheumatologic, cardiovascular, gastrointestinal, respiratory, renal, and ocular systems. Arthralgia, inflammatory myopathy with proximal or generalized weakness, and fibromyalgia may also occur as a result of this disease (7).

Various treatments for scleromyxedema have been reported such as corticosteroids, melphalan, thalidomide, IVIG, retinoids, extracorporeal photophoresis, and autologous stem cell transplantation, but a lack of knowledge of this disease’s etiology makes treatment difficult (8). Owing to the rarity of this entity, randomized, controlled clinical trials are not likely. There is no evidence of definitive guidelines on the best approach to treatment. In the past, monthly courses of melphalan, targeting the plasma cell dyscrasia, were often the therapy of choice. Melphalan can result in some clinical improvement. However, it has also been implicated in 30% of the deaths secondary to its induction of hematologic malignancies and septic complications (9). Our patient was administered with oral acitretin for 4 months and methylprednisolone with thalidomide for 6 months, respectively. Her cutaneous symptoms and systemic symptoms worsened over time. However, significant improvement was achieved after conversion to IVIG treatment.

Intravenous immunoglobulin was the preferred initial therapy, with relatively high efficacy on both skin and extracutaneous manifestations, possibly because of its immunomodulatory and antifibrotic effects (10). It is usually administered at a dose of 2 g/kg/mo, over a 4- to 5-day treatment cycle. Skin assessment should be performed every three cycles. If the clinical response is not satisfactory, or treatment efficacy diminishes over time, combining IVIG with systemic corticosteroids and thalidomide is suggested (4). In addition, long-term treatment is usually required for scleromyxedema to remain in remission. Our patient was successfully treated with six courses of IVIG per month. Their systemic and cutaneous symptoms showed significant improvement. We recommend that IVIG is administered every 6 weeks to maintain remission. Herein, we reported a case of scleromyxedema associated with paraproteinemia, in the treatment of which other therapies failed, successfully treated with IVIG. Prospective and comparative studies are still needed to confirm the efficacy and safety of IVIG treatments for this rare yet life-threatening disorder.

## Data availability statement

The original contributions presented in the study are included in the article/supplementary material. Further inquiries can be directed to the corresponding author.

## Ethics statement

Written informed consent was obtained from the Huashan Hospital Affiliated to Fudan University for the publication of any potentially identifiable images or data included in this article. The patients/participants provided their written informed consent to participate in this study. Written informed consent was obtained from the individual(s) for the publication of any potentially identifiable images or data included in this article.

## Author contributions

S-sW and Q-yC collected the clinical data and drafted the manuscript. L-hX was responsible for the study design. All authors read and approved the final manuscript.
